# Temporal Dissociation between Myeloperoxidase (MPO)-Modified LDL and MPO Elevations during Chronic Sleep Restriction and Recovery in Healthy Young Men

**DOI:** 10.1371/journal.pone.0028230

**Published:** 2011-11-30

**Authors:** Karim Zouaoui Boudjeltia, Brice Faraut, Maria José Esposito, Patricia Stenuit, Michal Dyzma, Pierre Van Antwerpen, Dany Brohée, Luc Vanhamme, Nicole Moguilevsky, Michel Vanhaeverbeek, Myriam Kerkhofs

**Affiliations:** 1 Laboratory of Experimental Medicine, Centre Hospitalo-Universitaire de Charleroi, André Vésale Hospital, Université Libre de Bruxelles, Montigny-le-Tilleul, Belgium; 2 Sleep Laboratory, Centre Hospitalo-Universitaire de Charleroi, André Vésale Hospital, Université Libre de Bruxelles, Montigny-le-Tilleul, Belgium; 3 Laboratory of Pharmaceutical Chemistry, Université Libre de Bruxelles, Brussels, Belgium; 4 Laboratory of Molecular Parasitology, Institute for Molecular Biology and Medicine, Université Libre de Bruxelles, Gosselies, Belgium; 5 Technology Transfer, University of Namur, Namur, Belgium; University of Pennsylvania School of Medicine, United States of America

## Abstract

**Objectives:**

Many studies have evaluated the ways in which sleep disturbances may influence inflammation and the possible links of this effect to cardiovascular risk. Our objective was to investigate the effects of chronic sleep restriction and recovery on several blood cardiovascular biomarkers.

**Methods and Results:**

Nine healthy male non-smokers, aged 22–29 years, were admitted to the Sleep Laboratory for 11 days and nights under continuous electroencephalogram polysomnography. The study consisted of three baseline nights of 8 hours sleep (from 11 pm to 7 am), five sleep-restricted nights, during which sleep was allowed only between 1 am and 6 am, and three recovery nights of 8 hours sleep (11 pm to 7 am). Myeloperoxidase-modified low-density lipoprotein levels increased during the sleep-restricted period indicating an oxidative stress. A significant increase in the quantity of slow-wave sleep was measured during the first recovery night. After this first recovery night, insulin-like growth factor-1 levels increased and myeloperoxidase concentration peaked.

**Conclusions:**

We observed for the first time that sleep restriction and the recovery process are associated with differential changes in blood biomarkers of cardiovascular disease.

## Introduction

The National Sleep Foundation report indicates that 43% of the American population sleep less than 7 hours on work days, compared with 30% during the weekend [1]. This report also shows that sleep duration increases during the week-end, creating a possible recovery effect after five consecutive working days of shorter sleep duration (20% of adults report sleeping less than 6 h/night). Increasing epidemiologic evidence indicates that sleep deprivation is associated with an increased risk of cardiovascular events [2]. In a laboratory study, Van Leeuwen *et al.* [3] showed that 5 nights of sleep restriction (4 h) increased the production of proinflammatory molecules, such as interleukin (IL)-1β, IL-6, IL-17 and high sensitivity C-reactive protein (hs-CRP). It is noteworthy that the levels of these molecules remained elevated after 2 nights of recovery sleep. Recently, we confirmed the interactions between sleep and the immune system [4]. An increase in peripheral blood leukocyte count was observed after acute sleep restriction (2 h of sleep), which persisted after a recovery night. In addition, a short midday nap prior to the recovery night or an extended night of recovery sleep normalized this increase. Interestingly, an increase in plasma myeloperoxidase (MPO), an enzyme that catalyzes oxidation reactions via the release of reactive halogenating and nitrating species, was also observed after acute sleep restriction.

It is well known that biomarkers of oxidative stress accurately reflect the presence of cardiovascular risk, the extent of cardiovascular disease (CVD), and cardiovascular outcome. MPO and modified low density lipoprotein (LDL) are examples of such biomarkers [5]. On this background, we studied leukocyte subsets, fibrinogen, hs-CRP, MPO [6]–[7], MPO-modified LDL (Mox-LDL) and IL-8 [8], in nine healthy subjects during a protocol of chronic sleep restriction and recovery for 11 consecutive days and nights.

## Results

### Effects of sleep restriction and recovery on sleep architecture

The amounts of stage 2 and REM sleep were significantly reduced during all sleep restricted nights vs. baseline [Chi-square (6) = 27.7, p<0.001 and Chi-square (6) = 30.3, p<0.001, respectively; Table 1] and vs. the 3^rd^ baseline night [Chi-square (6) = 14.4, p = 0.01 and Chi-square (6) = 30.2, p<0.001, respectively; Table S1]. During sleep restricted nights 1 to 4, there was a decrease in the percentage of stage 2 sleep with a concomitant increase in the percentage of slow-wave sleep (SWS) vs. baseline [Chi-square (6) = 15.7, p = 0.04 and Chi-square (6) = 8.4, p = 0.03, respectively] and vs. the 3^rd^ baseline night [Chi-square (6) = 8.0, p = 0.01 and Chi-square (6) = 18.4, p = 0.01, respectively]. On the first recovery night, there was a significant increase in the quantity of SWS when compared to baseline [Chi-square (6) = 15.1, p = 0.01] and to the 3^rd^ baseline night [Chi-square (6) = 11.4, p = 0.01]. Sleep efficiency was significantly higher from the 3^rd^ sleep restricted night to the first recovery night as compared to baseline [Chi-square (6) = 17.3, p = 0.004] and to the 3^rd^ baseline night [Chi-square (6) = 14.4, p = 0.01]. Total sleep time was reduced during the sleep restricted nights and increased during the first recovery night vs. baseline [Chi-square (6) = 17.6, p = 0.003] and vs. the 3^rd^ baseline night [Chi-square (6) = 22.0, p = 0.001].

**Table 1:** Sleep architecture at baseline and during sleep restricted and recovery nights.

	Baseline	Restriction 1	Restriction 2	Restriction 3	Restriction 4	Restriction 5	Recovery 1	Recovery 2	Recovery 3	*P* Value
Stage 1(min)	8.7(6.1-12.0)	8.0(3.5-10.5)	3.5(1.5-6.5)	2.0(1.5-5.0)	2.5(2.0-7.0)	3.0(1.5-8.5)	5.5(1.5-8.5)	9.5(7.0-14.5)	5.0(3.2-13.5)	0.17
Stage 2(min)	169.7(159.5-215.5)	94.5*(86.0-112.5)	85.5*(68.0-111.0)	100.5*(85.5-115.5)	93.0*(66.5-114.0)	114.5*(89.0-124.5)	165.0(124.5-205.5)	190.0(173.5-224.5)	196.0(155.2-211.7)	**< 0.001**
SWS(min)	124.0(99.0-152.6)	121.5(91.5-141.0)	119.0(87.5-142.0)	115.0(98.0-126.0)	117.5(94.0-132.0)	110.0(95.0-122.0)	163.0*(124.0-198.5)	129.5(102.5-160.5)	137.5(95.0-152.0)	**0.01**
REM(min)	100.0(87.6-109.25)	43.0*(38.5-52.5)	51.5*(50.0-70.0)	62.0*(40.0-71.5)	53.5*(46.5-70.5)	67.0*(59.0-72.5)	102.5(76.5-113.0)	97.5(87.5-112.0)	89.0(62.7-116.2)	**< 0.001**
Stage 1(%)	2.1(1.4-2.9)	2.9(1.4-3.8)	1.3(0.5-2.4)	0.7(0.5-1.8)	2.5(0.7-2.4)	1.0(0.5-2.3)	1.2(0.4-1.9)	2.2(1.6-3.1)	1.4(0.8-3.3)	0.12
Stage 2(%)	47.0(38.5-51.2)	34.7*(34.1-40.5)	31.9*(24.8-41.8)	37.1*(33.5-41.5)	32.8*(23.9-41.9)	43.0(30.5-45.0)	36.9(31.5-45.0)	42.9(38.7-51.5)	45.3(38.1-52.1)	**0.01**
SWS(%)	27.7(22.9-35.0)	45.5*(35.2-47.4)	45.7*(32.1-50.2)	43.0*(39.5-47.3)	41.9*(35.4-46.3)	39.4(35.5-50.8)	36.3(31.5-44.4)	29.8(23.3-35.6)	33.1(22.9-40.7)	**0.03**
REM(%)	24.2(21.4-25.9)	16.4*(15.2-18.7)	19.3(17.9-25.2)	23.0(16.1-25.9)	19.5(16.7-25.3)	24.4(20.2-25.7)	22.6(16.6-29.7)	22.2(19.7-26.0)	21.2(14.4-22.4)	**0.02**
Sleep efficiency (%)	88.7(85.3-90.6)	88.1(84.1-93.8)	91.3(89.1-93.6)	93.6*(88.9.-95.6)	94.3*(93.0-95.6)	95.9*(93.4-98.0)	93.4*(92.1-95.0)	90.8(89.0-94.8)	88.3(86.5-90.5)	**0.004**
Total sleep time(min)	419.3(396.0-421.2)	267.0*(252.0-280.0)	272.0*(260.5-280.0)	275.0*(273.5-284.0)	280.0*(252.0-280.0)	279.5*(271.0-288.0)	452.0*(444.0-456.2)	435.5(427.0-454.0)	418.5(403.0-425.0)	**0.003**

Values are shown as medians (25%–75% range). Baseline (average of the 3 baseline nights for each volunteer) was used as control and other nights as the comparative groups. Data comparisons with the baseline were performed using a Friedman Repeated Measures Analysis of Variance on Ranks and a Dunn's post-hoc test.

*Significant difference vs. baseline (*P*<0.05).

### Leukocyte, fibrinogen and hs-CRP

Leukocyte counts and leukocyte subsets (neutrophils, lymphocytes and monocytes) remained constant throughout the experiment (Table 2, Table S2). There were no statistically significant changes in the concentrations of hs-CRP or fibrinogen during the sleep restriction or recovery periods.

**Table 2:** Immune and inflammatory blood markers at baseline, and during sleep restriction and recovery periods.

	Baseline	Restriction 1	Restriction 3	Restriction 5	Recovery 1	Recovery 2	Recovery 3	*P* Value
Leukocytes(10^3^cells/μl)	6.77(5.58-7.32)	7.2(5.92-7.85)	6.86(6.47-7.43)	7.40(6.30-8.22)	7.10(5.95-7.76)	6.50(6.07-8.30)	6.70(5.97-7.90)	0.36
Neutrophils(10^3^cells/μl)	3.56(2.66-4.13)	3.76(2.96-4.63)	3.72(3.34-4.02)	3.96(3.28-4.34)	3.58(3.21-4.05)	3.79(2.70-4.57)	3.42(2.97-4.45)	0.19
Lymphocytes(10^3^cells/μl)	2.42(2.27-2.64)	2.35(1.93-2.59)	2.34(2.1-2.5)	2.44(2.0-2.6)	2.56(2.3-2.7)	2.37(2.1-4.1)	3.3(2.6-4.1)	0.62
Monocytes(10^3^cells/μl)	0.55(0.49-0.62)	0.61(0.53-0.72)	0.56(0.51-0.68)	0.61(0.51-0.72)	0.57(0.52-0.66)	0.65(0.49-0.74)	0.65(0.50-0.71)	0.18
Fibrinogen(mg/dl)	2.76(2.49-2.98)	2.95(2.71-3.07)	2.78(2.54-2.98)	2.71(2.41-2.94)	2.72(2.39-2.99)	2.74(2.47-2.95)	2.90(2.44-3.18)	0.13
Hs-CRP(mg/dl)	0.09(0.07-0.11)	0.10(0.06-0.14)	0.08(0.06-0.09)	0.08(0.05-0.09)	0.08(0.06-0.09)	0.06(0.06-0.09)	0.07(0.05-0.11)	0.11
Interleukin-8(pg/ml)	4.70(4.34-6.00)	6.36(4.33-6.00)	4.78(3.39-5.86)	4.68(4.03-6.91)	5.88(4.59-9.61)	6.60(3.75-10.457)	4.36(3.61-5.48)	0.11
ApoB (mg/dl)	74.1(71.7-89.4)	72.9(68.1-93.7)	71.6(64.4-93.2)	68.5(64.0-85.6)	68.9(64.5-85.9)	73.1(63.0-86.7)	66.1*(64.0-84.9)	**0.007**
Mox-LDL(µg/ml)	8.1(3.35-11.50)	12.3*(4.51-28.19)	14.6*(4.47-23.03)	10.3(4.40-11.92)	9.2(3.51-12.73)	8.8(3.47-14.19)	6.8(4.87-11.11)	**0.002**
IGF-1(pg/ml)	0.95(0.72-1.09)	0.88(0.69-1.23)	0.97(0.66-1.40)	0.95(0.76-1.22)	1.18*(0.83-1.38)	0.92(0.80-1.16)	0.87(0.80-1.08)	**0.03**

Values are shown as medians (25%–75% range). Baseline (average of the 3 baseline days for each volunteer) was used as control and other days as the comparative groups. Data comparisons with the baseline were performed using a Friedman Repeated Measures Analysis of Variance on Ranks and a Dunn's post-hoc test.

*Significant difference vs. baseline (*P*<0.05).

### MPO, Mox-LDL, IL-8 and IGF-1

The levels of Mox-LDL were significantly increased during the first and third days of sleep restriction compared to the baseline level [Chi-square (6) = 20.9, p = 0.002] (Table 2) and to the level after the 3^rd^ baseline night [Chi-square (6) = 20.52, p = 0.002; Table S2]. Interestingly, no change in ApoB concentration was observed during the sleep restricted phase. ApoB significantly decreased after the last night of recovery sleep compared to the baseline level [Chi-square (6) = 17.1 p = 0.007] and to the level after the 3^rd^ baseline night [Chi-square (6) = 16.54, p = 0.01].

**Figure 1:**
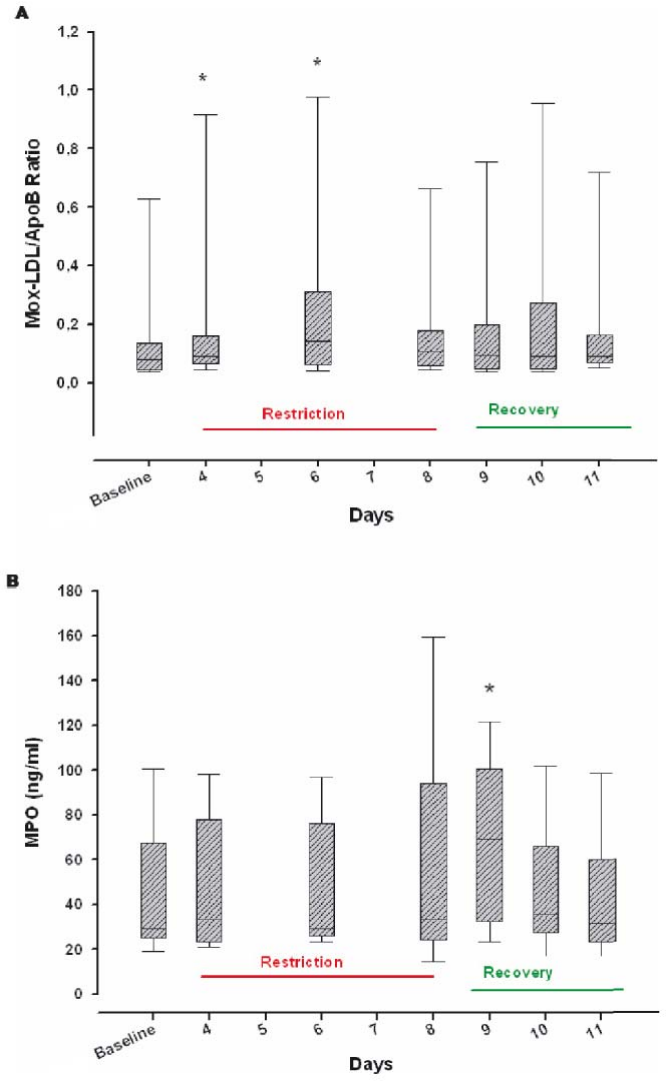
Myeloperoxidase (MPO)-modified low-density lipoprotein/apolipoprotein B (Mox-LDL/ApoB) ratio (A) and MPO (B) at baseline, and during sleep restriction and recovery periods. Values are shown as medians (25%–75% range). Baseline (average of the 3 baseline days for each volunteer) was used as control and other days as the comparative groups. Data comparisons with the baseline were performed using a Friedman Repeated Measures Analysis of Variance on Ranks and a Dunn's post-hoc test. * Significant difference vs. baseline (*P*<0.05).

The Mox-LDL/ApoB ratio (an estimation of the proportion of MPO modified-LDL in the bloodstream) was markedly increased during the 1^st^ and 3^rd^ days of sleep restriction compared to the baseline level [Chi-square (6) = 12.7, p = 0.04] (Fig. 1A) and to the level after the 3^rd^ baseline night [Chi-square (6) = 13.1, p = 0.03]. MPO concentration reached a peak after the first recovery night compared to the baseline concentration (69.3 [34.1–98.6] ng/ml versus 29.4 [25.3–57.2] ng/ml respectively, Chi-square (6) = 20.5, p = 0.002) (Fig. 1B) and to the concentration after the 3^rd^ baseline night [Chi-square (6) = 20.37, p = 0.002]. There was no significant change in IL-8 levels during the protocol compared to the baseline level [Chi-square (6) = 10.3, p = 0.11] or to the level after the 3^rd^ baseline night [Chi-square (6) = 11.9, p = 0.07]. IGF-1 increased significantly after the first recovery night compared to baseline [Chi-square (6) = 13.2, p = 0.03] and to the concentration after the 3^rd^ baseline night [Chi-square (6) = 13.92, p = 0.03].

## Materials and Methods

### Ethics statement

The protocol was approved by the Ethics Committee of the ISPPC Hospital and all volunteers gave their written informed consent. The volunteers received financial compensation for their participation in the study.

### Subjects

Nine healthy male non-smokers (aged 22–29 years, body mass index between 19 and 25) without neurological, psychiatric, cardiac, or endocrine disease participated in the experiment. The volunteers did not drink alcohol on a regular basis and were not taking any drugs. Each volunteer underwent a rigorous medical evaluation that included psychiatric and medical histories and screening laboratory tests (complete blood cell count, routine chemistry panel, and lipid profile). No sleep complaints were reported and sleep disorders were excluded on the basis of an interview and one night of polysomnographic recording.

### Experimental procedure

After two weeks of regular sleep-wake behavior with 8 h of sleep per night documented by actigraphic recordings and sleep diaries, the volunteers were admitted to the Sleep Laboratory for eleven nights and days. The study consisted of three baseline nights (sleeping from 11 pm to 7 am), five sleep restricted nights, during which sleep was allowed only between 1 am and 6 am, and three recovery nights of 8 hours sleep (11 pm to 7 am). During the study, the subjects were closely supervised and intake of any medication, alcohol, or xanthine derivatives (coffee, tea, chocolate, cola) was prohibited. Subjects received standard hospital meals of a maximum of 2500 calories/day with comparable proportions of nutrients (protein, fats, carbohydrates) across days and sessions. Controlled drinks and snacks were available during the sleep restricted nights until 11 pm. To monitor the state of alertness of the subjects and to ensure no sleep episodes occurred outside the permitted hours, continuous EEG, electrooculogram and electromyogram recordings were performed with an ambulatory device (Dream®, Medatec, Brussels, Belgium) with the following EEG derivations (C4/A1, C3/A2, *O2/A1*, O1/A2 F4/A1, F3/A2). During the study, volunteers were free to move within the unit carrying this ambulatory device, which was also used for the night recordings. Sleep recordings were scored visually in all subjects according to Rechtschaffen and Kales [9].

### Blood parameters

Fasting blood samples were obtained from an antecubital vein at 7:00 am after the 1^st^, 2^nd^ and 3^rd^ baseline nights, after the 1^st^, 3^rd^ and 5^th^ restricted nights, and after the 1^st^, 2^nd^ and 3^rd^ nights of sleep recovery with the volunteers comfortably seated in an armchair. Whole blood was collected in EDTA-treated tubes; serum samples were collected in vacuum tubes without anticoagulant; plasma samples were harvested in citrated vacuum tubes. CRP and apolipoprotein B (ApoB) were evaluated on SYNCHRON LX®. Fibrinogen was determined on a STA® automate (Stago, Parsippany, NJ). Leukocyte counts and subsets were determined on a CELL-DYN4000® hemocytometer (Abbott, Abbott Park, IL). The antibodies used for the measurement of Mox-LDL have been characterized previously [25]. Serum IL-8 concentrations were quantified using an ELISA test (Becton Dickinson®, Franklin Lakes, NJ) as were plasma MPO levels (Zentech®, Angleur, Belgium) and insulin-like growth factor (IGF)-1 levels (Diagnostic Systems Laboratories, Sinsheim, Germany).

### Statistics

Data were analyzed using the SigmaStat® 3.5 software (Systat®, San Jose, CA). Median values with range (25%–75%) are given because the data were not normally distributed (Kolmogorov-Smirnov) and we therefore used non-parametric tests with the Friedman Repeated Measures Analysis of Variance on Ranks test, which includes a chi-square test, being the most appropriate for our study design. This test displays the results of the chi-square, degrees of freedom and p value. The baseline (median of the mean values of the 3 baseline nights for each volunteer) or the 3^rd^ baseline values were used as the control and other days as the comparative groups. Data comparisons with the baseline or the 3^rd^ baseline values were performed using a Dunn's post-hoc test. A probability level of p<0.05 was considered statistically significant.

## Discussion

Our main findings were that Mox-LDL concentrations increased during chronic restricted sleep, indicating oxidative stress, and MPO concentrations peaked after the first recovery night. Moreover, there was a significant rebound of SWS during the first recovery night as compared to baseline. Although having a control group would certainly have improved the protocol design, there was good reproducibility of all measured blood and sleep parameters during the baseline nights indicating that they were not significantly influenced by the presence of the volunteers in the sleep laboratory. Moreover, when we compared the restriction and recovery night values with values from the 3^rd^ baseline night (with the first two baseline nights then considered as allowing participants to adapt to the laboratory setting as adaptation nights), the results were similar to those obtained using the average of the 3 baseline night values as the comparator.

Many studies have suggested that sleep duration plays a significant role in CVD [10]–[12]; however, the mechanisms involved have not been clearly elucidated. Epidemiological studies have identified a link between leukocyte counts and an increased risk of CVD [13]. Within the leukocyte sub-population, monocytes [14] and neutrophils [15] may influence the development of coronary heart disease through their ability to cause proteolytic and oxidative damage to coronary arteries. We previously observed in postmenopausal women that sleep restriction to four hours of sleep for three consecutive nights was associated with increased leukocyte, monocyte, and neutrophil counts, and total cholesterol and LDL-cholesterol (LDL-c) [16]. In a second study, using the same sleep restriction protocol but in healthy young men, only the neutrophil count was increased suggesting that sex differences may be a potential confounding factor on the effects of chronic sleep restriction although the age difference between the two studies also needs to be taken into account [17]. In the present study, there was a small but non-significant increase in neutrophil count during the sleep restricted days. Our previous results suggested that neutrophil count could be a very sensitive, early immunological marker of sleep loss, and may reflect the sleep debt [18]. Accordingly, the neutrophil response may be proportional to the intensity of sleep restriction. The moderate degree of sleep restriction in the present study was likely insufficient to cause the significant increase in neutrophil count that we previously observed in more severe, acute sleep restriction protocols [4], [17].

Similar to our present and earlier observations [16], [17], Haack et al [19] reported no significant change in hs-CRP levels after chronic sleep restriction with 12 consecutive days of 4 hours sleep per night. In contrast, Meier-Ewert et al. [20] observed an increase in hs-CRP levels during chronic sleep restriction of 4.2 hours per night for 10 days and after 88 hours of total sleep deprivation, and Frey et al. [21] reported a decrease in hs-CRP following one night of total sleep deprivation. Multiple blood samplings measured from an indwelling catheter were not performed to avoid any confounding effects of local inflammation from the blood drawing procedure as has previously been reported in the production of local inflammatory cytokines [22]. Potential confounding effects of a circadian shift could, therefore, have contributed to the unchanged hs-CRP we observed with a single daily blood sample, but hs-CRP protein levels have been described as being relatively stable over a 24-hour period and not displaying a circadian rhythm [23]. The reasons for the apparent discrepancies between our study and other similar chronic sleep restriction studies, including that by van Leeuwen et al. [3], are not clear. Some potentially causative differences between our two protocols remain in terms of the greater intensity of the sleep restriction in the study by van Leeuwen at al. (4 hours of sleep for 5 consecutive nights), as well as the timing of the restriction period. Nevertheless, both studies reported increases in the cardiovascular risk biomarkers, i.e., hs-CRP and MPO, which persisted or peaked after the recovery nights. An additional potential confounding factor could be that during the sleep restricted phase, blood was sampled after subjects had been awake for 1 hour, whereas during baseline periods, blood was sampled right after waking the subjects. An argument suggesting that this factor did not significantly influence the levels of Mox-LDL production and MPO levels is that there was no change in MPO or ApoB (the protein modified by the MPO) levels between the baseline nights (blood sampling at 7 am right after waking) and the restriction phase (blood sampling at 7 am, 1 hour after waking). A slight decrease in ApoB (previously reported to display low diurnal variability) was measured during the recovery period, possibly induced by the rise in blood IGF-1, a negative regulator of ApoB expression in healthy men [24], [25].

During our study, there was a temporal discordance between the Mox-LDL/ApoB ratio and the increase in MPO concentrations. Indeed, the Mox-LDL/ApoB ratio increased after the first night of sleep restriction in contrast to MPO, which increased after the first recovery night. The capture antibody we used for this analysis reacts only with LDL modified by the MPO-H_2_O_2_-Cl^−^ system [26]. Previously, we showed that the membrane-bound nicotinamide-adenine-dinucleotide phosphate (NADPH) oxidase of endothelial cells played a central role in Mox-LDL production, even at constant MPO concentrations [27]. The O^−^_2_ generated by membrane NADPH oxidase is the starting substrate for MPO to catalyze the production of H_2_O_2_ to form HOCl. Catecholamines have been shown to activate NADPH-dependent vascular oxidases [28]. Irwin et al [29] reported that loss of sleep or disordered sleep increased blood catecholamine concentrations, suggesting that this mechanism could be one explanation for the increased Mox-LDL during sleep restriction without changes in MPO. Of interest is the increase in the Mox-LDL/ApoB ratio range during the restriction phase. This may suggest that some subjects are more sensitive than others to the sleep restriction procedure. In addition, why did the Mox-LDL/ApoB ratio decrease at the end of the sleep restriction phase? One explanation could be that the modified LDL increases the expression of scavenger receptors inducing a secondary clearance of Mox-LDL from the blood as previously demonstrated [30].

Our results showed that the first recovery night was associated with an increased amount of SWS, concomitant with the presence of a peak in MPO level. We can only speculate on the reasons underlying this observation. Spiegel et al [31] reported a tight association between the amount of growth hormone secreted and the amount of SWS during sleep recovery. Secreted growth hormone stimulates production by the liver of circulating blood IGF-1 which displays immune and inflammatory regulatory properties [32], [33]. IGF-1 is able to induce degranulation of azurophilic granules (the MPO content) by peripheral blood mononuclear cells [34]. Van Leeuwen et al. [35] reported that the recovery period following chronic sleep restriction was associated with a significant increase in serum IGF-1 concentrations. We also observed a significant IGF-1 peak after the first recovery night, which may, in part, explain the peak in MPO seen after this recovery night.

What could be the biological effects of the variations in MPO and Mox-LDL? Clinical studies have reported associations between blood MPO levels and increased cardiovascular events [7], [36]. In our study, an increase was observed only after the first recovery night but potentially inducing a short-term oxidative effect. Indeed, we previously showed that MPO increased production of reactive oxygen species (ROS) on endothelial cells after 30 min of exposure [37]. In another study, we demonstrated that, in contrast to copper–oxidized LDLs (ox-LDL), Mox-LDL (more physiologically relevant) induced a greater production of ROS on human monocytes after 6 hours of exposure [38]. Only Mox-LDL-induced ROS production was dependent on cytosolic phospholipase A2.

In conclusion, our results show for the first time that the recovery process after chronic sleep restriction is coupled to changes in blood biomarkers that have been associated with cardiovascular risk. Further sleep studies that modulate the intensity and duration of the sleep restriction and recovery processes are required to better understand this phenomenon. An additional issue that needs further investigation is whether there is a difference in the response of cardiovascular risk markers to sleep restriction in men and women. Sex-specific differences in the inflammatory response were reported in a laboratory study following one night of partial sleep loss in same age populations [39]. Emerging evidence from epidemiological studies also indicates that women may have different chronic responses to sleep duration compared to men, and our data need, therefore, to be confirmed in women [40].

## Supporting Information

Table S1 **Sleep architecture at 3^rd^ baseline night and during sleep restricted and recovery nights.** Values are shown as medians (25%–75% range). Baseline 3 (the 3^rd^ baseline night) was used as control and other nights as the comparative groups. Data comparisons with baseline 3 were performed using a Friedman Repeated Measures Analysis of Variance on Ranks and a Dunn's post-hoc test. * Significant difference vs. baseline 3 (*P*<0.05).
(DOC)

Table S2 **Immune and inflammatory blood markers after the 3^rd^ baseline night and during sleep restriction and recovery periods.** Values are shown as medians (25%–75% range). Baseline 3 (the 3^rd^ baseline day) was used as control and other days as the comparative groups. Data comparisons with baseline 3 were performed using a Friedman Repeated Measures Analysis of Variance on Ranks and a Dunn's post-hoc test. * Significant difference vs. baseline 3 (*P*<0.05).
(DOC)
