# Supplementary material for: Temporal Dissociation between Myeloperoxidase (MPO)-Modified LDL and MPO Elevations during Chronic Sleep Restriction and Recovery in Healthy Young Men
Source: PLoS One. 2011 Nov 30;6(11):e28230. doi: 10.1371/journal.pone.0028230 (PMC3227655; doi:10.1371/journal.pone.0028230)
Supplement: Table S1 — Sleep architecture at 3rd baseline night and during sleep restricted and recovery nights. Values are shown as medians (25%–75% range). Baseline 3 (the 3rd baseline night) was used as control and other nights as the comparative groups. Data comparisons with baseline 3 were performed using a Friedman Repeated Measures Analysis of Variance on Ranks and a Dunn's post-hoc test. * Significant difference vs. baseline 3 (P<0.05). (DOC) [file pone.0028230.s001.doc]

|  | Baseline 3 | Restriction 1 | Restriction 2 | Restriction 3 | Restriction 4 | Restriction 5 | Recovery 1 | Recovery 2 | Recovery 3 | *P* Value |
| --- | --- | --- | --- | --- | --- | --- | --- | --- | --- | --- |
| Stage 1  (min) | 10.0  (5.7-18.0) | 8.0  (3.5-10.5) | 3.5  (1.5-6.5) | 2.0  (1.5-5.0) | 2.5  (2.0-7.0) | 3.0  (1.5-8.5) | 5.5  (1.5-8.5) | 9.5  (7.0-14.5) | 5.0  (3.2-13.5) | 0.13 |
| Stage 2  (min) | 193.0  (159.2-207.2) | 94.5 *  (86.0-112.5) | 85.5 *  (68.0-111.0) | 100.5 *  (85.5-115.5) | 93.0 *  (66.5-114.0) | 114.5 *  (89.0-124.5) | 165.0  (124.5-205.5) | 190.0  (173.5-224.5) | 196.0  (155.2-211.7) | **0.01** |
| SWS  (min) | 119.0  (103.2-149.2) | 121.5  (91.5-141.0) | 119.0  (87.5-142.0) | 115.0  (98.0-126.0) | 117.5  (94.0-132.0) | 110.0  (95.0-122.0) | 163.0 *  (124.0-198.5) | 129.5  (102.5-160.5) | 137.5  (95.0-152.0) | **0.01** |
| REM  (min) | 109.0  (91.7-122.5) | 43.0 *  (38.5-52.5) | 51.5 *  (50.0-70.0) | 62.0 *  (40.0-71.5) | 53.5 *  (46.5-70.5) | 67.0 *  (59.0-72.5) | 102.5  (76.5-113.0) | 97.5  (87.5-112.0) | 89.0  (62.7-116.2) | **< 0.001** |
| Stage 1  (%) | 2.3  (1.3-4.2) | 2.9  (1.4-3.8) | 1.3  (0.5-2.4) | 0.7  (0.5-1.8) | 2.5  (0.7-2.4) | 1.0  (0.5-2.3) | 1.2  (0.4-1.9) | 2.2  (1.6-3.1) | 1.4  (0.8-3.3) | 0.10 |
| Stage 2  (%) | 45.9  (37.8-53.1) | 34.7 *  (34.1-40.5) | 31.9 *  (24.8-41.8) | 37.1 *  (33.5-41.5) | 32.8 *  (23.9-41.9) | 43.0  (30.5-45.0) | 36.9  (31.5-45.0) | 42.9  (38.7-51.5) | 45.3  (38.1-52.1) | **0.01** |
| SWS  (%) | 25.3  (20.0-33.2) | 45.5 *  (35.2-47.4) | 45.7 *  (32.1-50.2) | 43.0 *  (39.5-47.3) | 41.9 *  (35.4-46.3) | 39.4 *  (35.5-50.8) | 36.3  (31.5-44.4) | 29.8  (23.3-35.6) | 33.1  (22.9-40.7) | **0.03** |
| REM  (%) | 27.0  (22.1-30.1) | 16.4 *  (15.2-18.7) | 19.3  (17.9-25.2) | 23.0  (16.1-25.9) | 19.5  (16.7-25.3) | 24.4  (20.2-25.7) | 22.6  (16.6-29.7) | 22.2  (19.7-26.0) | 21.2  (14.4-22.4) | **0.03** |
| Sleep efficiency (%) | 89.9  (87.7-92.3) | 88.1  (84.1-93.8) | 91.3  (89.1-93.6) | 93.6 *  (88.9.-95.6) | 94.3 *  (93.0-95.6) | 95.9 *  (93.4-98.0) | 93.4 *  (92.1-95.0) | 90.8  (89.0-94.8) | 88.3  (86.5-90.5) | **0.01** |
| Total sleep time  (min) | 430.0  (414.2-442.2) | 267.0 *  (252.0-280.0) | 272.0 *  (260.5-280.0) | 275.0 *  (273.5-284.0) | 280.0 *  (252.0-280.0) | 279.5 *  (271.0-288.0) | 452.0 *  (444.0-456.2) | 435.5  (427.0-454.0) | 418.5  (403.0-425.0) | **0.001** |

**Table S1**
