# Supplementary material for: Temporal Dissociation between Myeloperoxidase (MPO)-Modified LDL and MPO Elevations during Chronic Sleep Restriction and Recovery in Healthy Young Men
Source: PLoS One. 2011 Nov 30;6(11):e28230. doi: 10.1371/journal.pone.0028230 (PMC3227655; doi:10.1371/journal.pone.0028230)
Supplement: Table S2 — Immune and inflammatory blood markers after the 3rd baseline night and during sleep restriction and recovery periods. Values are shown as medians (25%–75% range). Baseline 3 (the 3rd baseline day) was used as control and other days as the comparative groups. Data comparisons with baseline 3 were performed using a Friedman Repeated Measures Analysis of Variance on Ranks and a Dunn's post-hoc test. * Significant difference vs. baseline 3 (P<0.05). (DOC) [file pone.0028230.s002.doc]

|  | Baseline 3 | Restriction 1 | Restriction 3 | Restriction 5 | Recovery 1 | Recovery 2 | Recovery 3 | *P* Value |
| --- | --- | --- | --- | --- | --- | --- | --- | --- |
| Leukocytes  (10^3^cells/μl) | 7  (5.0.6-8) | 7.2  (5.92-7.85) | 6.86  (6.47-7.43) | 7.40  (6.30-8.22) | 7.10  (5.95-7.76) | 6.50  (6.07-8.30) | 6.70  (5.97-7.90) | 0.9 |
| Neutrophils  (10^3^cells/μl) | 3.71  (2.82-4.30) | 3.76  (2.96-4.63) | 3.72  (3.34-4.02) | 3.96  (3.28-4.34) | 3.58  (3.21-4.05) | 3.79  (2.70-4.57) | 3.42  (2.97-4.45) | 0.82 |
| Lymphocytes  (10^3^cells/μl) | 2.53  (2.15-2.65) | 2.35  (1.93-2.59) | 2.34  (2.1-2.5) | 2.44  (2.0-2.6) | 2.56  (2.3-2.7) | 2.37  (2.1-4.1) | 3.3  (2.6-4.1) | 0.13 |
| Monocytes  (10^3^cells/μl) | 0.50  (0.40-0.67) | 0.61  (0.53-0.72) | 0.56  (0.51-0.68) | 0.61  (0.51-0.72) | 0.57  (0.52-0.66) | 0.65  (0.49-0.74) | 0.65  (0.50-0.71) | 0.66 |
| Fibrinogen  (mg/dl) | 2.64  (2.59-3.04) | 2.95  (2.71-3.07) | 2.78  (2.54-2.98) | 2.71  (2.41-2.94) | 2.72  (2.39-2.99) | 2.74  (2.47-2.95) | 2.90  (2.44-3.18) | 0.13 |
| Hs-CRP  (mg/dl) | 0.10  (0.05-0.11) | 0.10  (0.06-0.14) | 0.08  (0.06-0.09) | 0.08  (0.05-0.09) | 0.08  (0.06-0.09) | 0.06  (0.06-0.09) | 0.07  (0.05-0.11) | 0.06 |
| Interleukin-8  (pg/ml) | 4.27  (3.49-5.08) | 6.36  (4.33-6.00) | 4.78  (3.39-5.86) | 4.68  (4.03-6.91) | 5.88  (4.59-9.61) | 6.60  (3.75-10.457) | 4.36  (3.61-5.48) | 0.06 |
| ApoB (mg/dl) | 76.6  (72-89.4) | 72.9  (68.1-93.7) | 71.6  (64.4-93.2) | 68.5  (64.0-85.6) | 68.9  (64.5-85.9) | 73.1  (63.0-86.7) | 66.1 *  (64.0-84.9) | **0.01** |
| Mox-LDL  (µg/ml) | 7.8  (3.49-14.34) | 12.3 *  (4.51-28.19) | 14.6 *  (4.47-23.03) | 10.3  (4.40-11.92) | 9.2  (3.51-12.73) | 8.8  (3.47-14.19) | 6.8  (4.87-11.11) | **0.002** |
| IGF-1  (pg/ml) | 0.95  (0.75-1.14) | 0.88  (0.69-1.23) | 0.97  (0.66-1.40) | 0.95  (0.76-1.22) | 1.18 *  (0.83-1.38) | 0.92  (0.80-1.16) | 0.87  (0.80-1.08) | **0.03** |
| MPO (ng/ml) | 29.4  (27.3-48.01) | 33.6  (23.1-70.4) | 29.4  (26.7-72.7) | 33.6  (24.6-90.7) | 69.3*  (38.4-98.6) | 35.7  (28.3-54.1) | 31.5  (24.1-48.9) | **0.002** |
| Mox-LDL/ApoB | 0.07  (0.04-0.14) | 0.09*  (0.06-0.14) | 0.14*  (0.06-0.24) | 0.10  (0.06-0.17) | 0.09  (0.05-0.17) | 0.09  (0.05-0.21) | 0.09  (0.06-0.14) | **0.03** |

**Table S2**
